# Serum levels of inflammatory cytokines in Rift Valley fever patients are indicative of severe disease

**DOI:** 10.1186/s12985-015-0392-3

**Published:** 2015-10-06

**Authors:** Petrus Jansen van Vuren, Sharon Shalekoff, Antoinette A. Grobbelaar, Brett N. Archer, Juno Thomas, Caroline T. Tiemessen, Janusz T. Paweska

**Affiliations:** Centre for Emerging and Zoonotic Diseases, National Institute for Communicable Diseases division of the National Health Laboratory Service, Sandringham, South Africa; Department of Microbiology and Plant Pathology, University of Pretoria, Pretoria, South Africa; Centre for HIV and STIs, National Institute for Communicable Diseases division of the National Health Laboratory Service, Sandringham, South Africa; Outbreak Response Unit, Division of Public Health, Surveillance and Response, National Institute for Communicable Diseases division of the National Health Laboratory Service, Sandringham, South Africa; Faculty of Health Sciences, School of Pathology, University of the Witwatersrand, Johannesburg, South Africa

**Keywords:** Rift Valley fever, Inflammation, Pathogenesis

## Abstract

**Background:**

Rift Valley fever (RVF) is a mosquito-borne viral zoonosis affecting domestic and wild ruminants, camels and humans. Outbreaks of RVF are characterized by a sudden onset of abortions and high mortality amongst domestic ruminants. Humans develop disease ranging from a mild flu-like illness to more severe complications including hemorrhagic syndrome, ocular and neurological lesions and death. During the RVF outbreak in South Africa in 2010/11, a total of 278 human cases were laboratory confirmed, including 25 deaths. The role of the host inflammatory response to RVF pathogenesis is not completely understood.

**Methods:**

Virus load in serum from human fatal and non-fatal cases was determined by standard tissue culture infective dose 50 (TCID_50_) titration on Vero cells. Patient serum concentration of chemokines and cytokines involved in inflammatory responses (IL-8, RANTES, CXCL9, MCP-1, IP-10, IL-1β, IL-6, IL-10, TNF and IL-12p70) was determined using cytometric bead assays and flow cytometry.

**Results:**

Fatal cases had a 1-log_10_ higher TCID_50_/ml serum concentration of RVF virus (RVFV) than survivors (*p* < 0.05). There were no significant sequence differences between isolates recovered from fatal and non-fatal cases. Chemokines and pro- and anti-inflammatory cytokines were detected at significantly increased (IL-8, CXCL9, MCP-1, IP-10, IL-10) or decreased (RANTES) levels when comparing fatal cases to infected survivors and uninfected controls, or when comparing combined infected patients to uninfected controls.

**Conclusions:**

The results suggest that regulation of the host inflammatory responses plays an important role in the outcome of RVFV infection in humans. Dysregulation of the inflammatory response contributes to a fatal outcome. The cytokines and chemokines identified in this study that correlate with fatal outcomes warrant further investigation as markers for disease severity.

## Background

Rift Valley fever (RVF) is a mosquito-borne zoonotic disease endemic to Africa, the Arabian Peninsula and islands in the Indian ocean off the east coast of Africa [1, 2]. RVF virus (RVFV), a member of the *Phlebovirus* genus in the *Bunyaviridae* family, infects domestic livestock, wild ruminants and humans. Humans acquire infection through the bite of infected mosquitoes or from contact with infected tissues or body fluids [3, 4]. High mortality rates have been reported in newborn and juvenile animals, while abortion storms are a prominent feature of RVF outbreaks in domesticated livestock [1]. Infected humans develop symptoms ranging from mild, flu-like illness to severe hemorrhagic fever, encephalitis and death in a small proportion of individuals [5, 6].

The non-structural protein encoded by the small (S) segment of the virus, NSs, has been implicated as the major virulence factor. It counteracts antiviral effects of the host type 1 interferon response through blocking the assembly of the transcription factor TFIIH, blocking IFN-α/β transcription through interaction with host SAP30 protein and post-transcriptional downregulation of protein kinase R [7–14]. The 14-kDa non-structural protein encoded by the medium (M) segment, NSm, has been shown to suppress virus induced apoptosis, and therefore it is believed that virulence of RVFV is under polygenic control [15, 16]. There are no commercially available RVF vaccines or therapeutics for humans, and the available livestock vaccines require multiple doses or have adverse effects [17, 18]. However, various vaccine candidates and experimental therapeutics were recently developed and tested in animal models [19–28].

The contributory role of human inflammatory responses to pathogenesis of RVFV infection has not been intensively studied largely due to lack of clinical material. We previously studied the expression of genes involved in innate and adaptive immunity after infection of BALB/c mice with a lethal dose of RVFV, compared to similar infection of mice protected by anti-NP immunity [29]. Various genes involved in pro-inflammatory responses and with pro-apoptotic effects were upregulated, and anti-apoptotic genes downregulated in infected mice with lethal outcome when compared to surviving mice. However, improperly timed high expression levels of interleukin-10 (IL-10), an anti-inflammatory immunosuppressive cytokine, in mice with a lethal outcome compared to survivors indicated an imbalance in the regulation of the inflammatory response, possibly contributing to pathogenesis and/or immune evasion early after infection [29]. A different study measuring actual protein concentration in samples from C57BL/6 mice infected with virulent (ZH-501) and attenuated RVFV (MP-12 vaccine strain) reached the same conclusion, with inflammatory cytokines significantly elevated in serum, liver and spleen of ZH-501 infected mice (100 % lethality) compared to those infected with MP-12 (100 % survival). In addition, a strong pro-inflammatory and anti-apoptotic response was detected in the brains of ZH-501 compared to MP-12 infected mice, which could explain development of neurological disease [30].

Contrary to the abovementioned studies in mice which showed a correlation between a pro-inflammatory response and lethal outcome, a recent study of human serum collected during the 2000/01 RVF outbreak in Saudi Arabia from laboratory confirmed cases found the opposite correlation. A total of 26 human sera, 6 from fatal and 20 from non-fatal cases were tested using a multiplex assay to determine serum concentration of 39 cytokines [31]. The investigators showed significant differences in concentrations of five soluble factors between fatal and non-fatal cases. Levels of the chemokine GRO (Growth related oncogene; CXCL1) and soluble CD40L (CD154) were significantly elevated in non-fatal vs fatal cases, while the contrary was true for cytokines IL-1α (proinflammatory) and IL-10 (immunosuppressive) as well as IL-1RA (a natural inhibitor that through binding the IL-1R counters the proinflammatory effects of IL-1α and IL-1β). The investigators concluded from their data that an overall proinflammatory response may be associated with patient survival. The ability of infected individuals to effectively control replication of RVFV, which is likely affected by various factors including immune competence, underlying conditions, co-infections, host genetics and overall health, contributes to a good prognosis. This is evidenced by data showing a clear correlation between virus concentration in serum and disease outcome, with virus replicating on to an average of 2.3 logs (TCID_50_) higher in fatal cases compared to non-fatal cases [32].

In this study we evaluated the role of chemokines and inflammatory cytokines in RVF pathogenicity by testing stored serum from patients who became infected during the 2010/11 outbreak of RVF in South Africa. Our results indicate that individual host innate response to infection, specifically inflammation, is likely an important contributing factor to pathogenesis. The up- or downregulation of certain inflammatory cytokines and chemokines correlated with either a good prognosis or a lethal outcome of infection. The data presented in our study further demonstrate the contributory role of dysregulated inflammatory responses in host individuals to RVF pathogenesis.

## Results

### Clinical data

Patients presented with non-specific symptoms such as fever, myalgia, malaise and headache. More detailed clinical information was available only for a portion of the fatal cases (*n* = 18). The information is summarized in Table 1. Not all of these cases were included in the virus titer and/or cytokine analysis due to insufficient sample volume. A total of 12 of the 18 fatal cases had severely raised liver enzymes (either one or both ALT and AST) on admission to hospital (liver enzymes could not be measured in two cases due to poor sample quality or insufficient volume); 12 had raised urea and creatinine; 16 had thrombocytopenia; 14 presented with hemorrhagic symptoms and four presented with encephalitis. Of note, three of the four fatal cases that presented with encephalitis were HIV positive (case submission data). Of the four, two presented only with encephalitis, one with additional thrombocytopenia and the last also with hepatitis, renal failure, thrombocytopenia and hemorrhagic syndrome.

**Table 1:** Clinical laboratory values for the individual fatal cases for which these values were available from the referring hospital

Sample ID	ALT (U/L)	Elevated/ low (normal ALT range 10–40)	AST (U/L)	Elevated /low (normal AST range 10–40)	Platelets (× 10^9^/L)	Elevated /low (normal platelet range 140–420)	White cell count (× 10^9^/L)	Elevated /low (normal white cell range 4–11)	Urea (mmol/L)	Elevated/low (normal urea range 2.5–6.7)	Creatinine (μmol/L)	Elevated/low (normal creatinine range 57–113)	Included in cytokine analysis?
SPU100/10	6397	Elevated	13149	Elevated	54	Low	n.a.	n.a.	n.a.	n.a.	n.a.	n.a.	Yes
SPU105/10	2965	Elevated	6280	Elevated	50	Low	6.07	Normal	4.9	Normal	96	Normal	Yes
SPU59/10	3148	Elevated	6938	Elevated	113	Low	12.6	Elevated	15.5	Elevated	504	Elevated	Yes
SPU97/10	1912	Elevated	7824	Elevated	13	Low	1.98	Low	22.3	Elevated	385	Elevated	Yes
SPU86/10	8007	Elevated	17719	Elevated	40	Low	6.23	Normal	16.2	Elevated	460	Elevated	Yes
SPU162/10	2906	Elevated	6424	Elevated	9	Low	7.91	Normal	34.1	Elevated	711	Elevated	Yes
SPU68/10	2991	Elevated	4742	Elevated	2	Low	9.11	Normal	59	Elevated	1421	Elevated	Yes
SA1372/10	4710	Elevated	9130	Elevated	13	Low	13.76	Elevated	20.9	Elevated	66.2	Normal	Yes
SA1151/10	3079	Elevated	913	Elevated	96	Low	14.28	Elevated	12.6	Elevated	501	Elevated	Yes
SA1457/10b	8353	Elevated	n.a.	n.a.	36	Low	8.96	Normal	16.9	Elevated	188	Elevated	Yes
SA979/10	30	Normal	87	Elevated	27	Low	16.85	Elevated	54.3	Elevated	2031	Elevated	Yes
SA1598/10^a^	37	Normal	108	Elevated	49	Low	4.91	Normal	3.3	Normal	55	Low	Yes
SA744/10	3359	Elevated	8192	Elevated	8	Low	6.16	Normal	22.6	Elevated	286	Elevated	Yes
SA1245/10^a^	39	Normal	126	Elevated	279	Normal	10.32	Normal	11.5	Elevated	66	Normal	No
SA559/10	n.a.	n.a.	n.a.	n.a.	56	Low	2	Low	n.a.	n.a.	n.a.	n.a.	No
SA663/10	n.a.	n.a.	n.a.	n.a.	65	Low	16.6	Elevated	15.9	Elevated	560	Elevated	No
SA596/10	10000	Elevated	14000	Elevated	8	Low	6	Normal	n.a.	n.a.	647	Elevated	No
SA558/10^a^	46	Elevated	107	Elevated	209	Normal	6.2	Normal	7.1	Elevated	109	Normal	No

*ALT* alanine transferase, *AST* aspartate transferase. The asterisk (^a^) indicates the three HIV positive cases, which presented with encephalitis. The hash sign (b) indicates the case which presented with encephalitis but with unknown HIV status

### Serum RVF virus titers

**Fig. 1:**
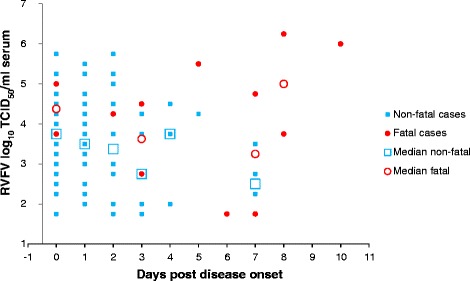
Individual and average RVFV viral loads in serum over time. Individual viral loads in serum are indicated per each time point post onset of disease: *blue squares* indicate individual non-fatal cases and *red dots* indicate individual fatal cases. Median viral loads at each time point are indicated by *open blue squares* (non-fatal) and *red circles* (fatal). Certain days are represented by only one data point per data set, in which case no medians are shown (days 5, 6, 10). Maximum two samples for respective days were available from fatal cases, thus averages are shown and not medians

Recent infection with RVF virus was confirmed in 278 cases, of which 25 were fatal, by one or more of the following methods: RT-PCR, IgM ELISA, virus isolation. Of the 25 fatal cases, virus titration was performed on 13 samples only due to limited volume (*n* = 8), and a portion of fatal cases were sampled only after the viremic stage (*n* = 4). Of the remaining 253 non-fatal RVF cases, virus was titrated from 85 samples. The remaining samples (*n* = 168) were excluded either due to insufficient sample volume or because the patients were no longer viremic at the time of blood collection. Due to insufficient number of samples taken on individual days post disease onset, statistical significance of differences in individual viral loads was calculated on the overall averages spanning all days post onset in fatal and non-fatal cases. The median serum viral loads, with confidence intervals and p-values to denote statistical significance, were determined. Individual serum viral loads are shown in Fig. 1. Blood was collected from fatal cases on average (median) 5 days post disease onset (range 0 – 10) and yielded a median viral load of log_10_ 4.5 TCID_50_/ml serum (log_10_ 3.7 – 5.3 at 95 % confidence). Blood collection from non-fatal cases occurred on average (median) 1 day post onset (range 0 – 7) and yielded a median viral load 1-log lower than that observed for fatal cases (log_10_ 3.5 TCID_50_/ml serum, range log_10_ 2.9 – 4.1 at 95 % confidence). The differences between fatal and non-fatal cases of collection post disease onset (*p* = 0.002) and viral load (*p* = 0.04) were statistically significant.

### Virus whole genome sequences

There were no significant nucleic acid sequence differences between isolates from fatal (*n* = 6) and non-fatal (*n* = 6) cases.

### Serum cytokine concentrations

From a total of 25 fatal cases, we performed cytometric bead array (CBA) cytokine analysis only on 19 samples [collected on average (median) 5 days post onset, range 0–10]. The six excluded samples could not be tested due to insufficient sample volume. A total of 33 serum samples from non-fatal cases were selected to represent the closest possible match to days post onset in fatal cases (and based on available volume) from the outbreak serum panel [collected on average (median) 2 days post onset, range 0 – 13]. A total of eight serum samples from healthy and RVF serologically negative (IgM and IgG, results not shown) individuals sampled during a post-outbreak survey were used as negative controls (representing healthy uninfected persons).

**Fig. 2:**
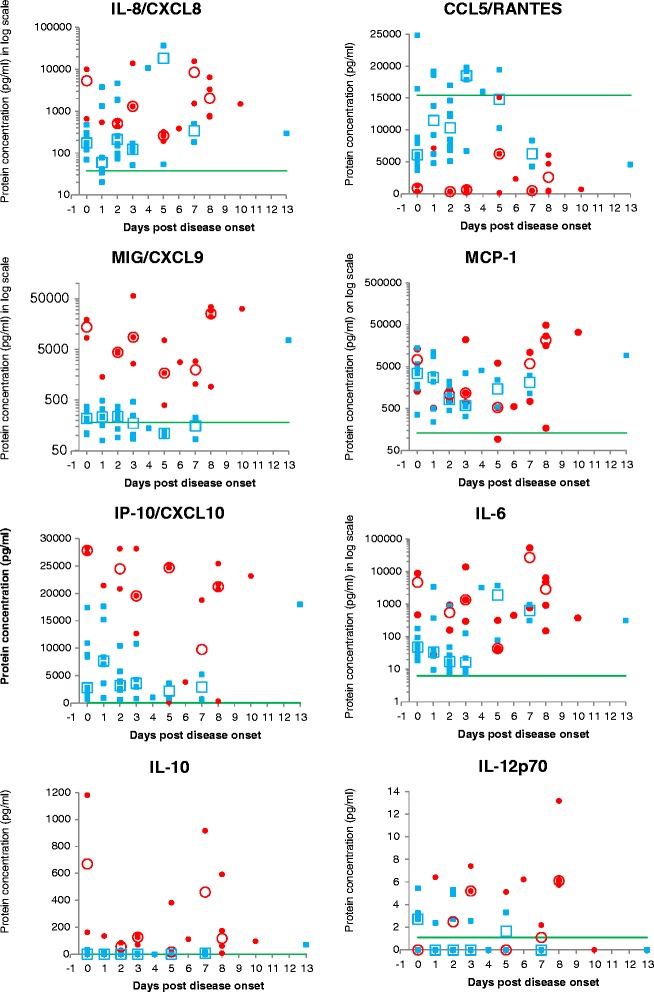
Individual and average cytokine concentrations in serum over time. Individual cytokine concentrations in serum are indicated per each time point post onset of disease: *blue squares* indicate individual non-fatal cases and *red dots* indicate individual fatal cases. Average concentrations at each time points are indicated by *open blue squares* (non-fatal) and *red circles* (fatal). The continuous *green line* indicates the average concentration in negative control samples (RVF naïve individuals) and thus has no bearing on the days indicated on the x-axis. Certain days are represented by only one data point per data set, in which case no medians are shown (days 1, 4, 6, 10 and 13). For days where less than three samples were available per dataset, averages are shown instead of medians. Note: because of the 1:4 dilution of serum for the cytokine analysis, a “0 pg/ml” value represents a limit of detection rather than absolute absence of the detected protein

Due to the same limitations as for measuring the viral loads, statistical significance of differences in cytokine responses were initially calculated using the overall averages spanning all days post disease onset. The results from individual cases are shown along with averages for specific days, in Table 2 and Fig. 2. For those cytokines and chemokines showing significant differences when analyzed using the overall data, the results from fatal and non-fatal cases were subsequently grouped in two groups according to early (days 0 – 3) and later periods (days 4 – 13) after disease onset and analyzed again between these two groups. The detection of serum tumor necrosis factor (TNF) and the p70 active heterodimer of interleukin-12 (IL-12p70) was very low and intermittent in all sample groups. The overall average concentration of interleukin-1-beta (IL-1β) was similar in all three groups, and in general detected at low concentrations. Serum levels of monokine-induced-by-gamma-interferon (CXCL9/MIG), interferon gamma-induced protein 10 (CXCL10/IP-10) and interleukin-10 (IL-10) were significantly increased in samples from fatal cases relative to survivors and controls using the overall data. When comparing early samples (day 0 – 3; *p* = 0.08) and later samples (day 4 – 13; *p* = 0.03) from fatal cases to non-fatal cases, the differences in CXCL9/MIG concentration were not statistically significant. CXCL10/IP-10 was significantly increased in early samples (*p* < 0.0001) from fatal cases compared to early samples from non-fatal cases, but not in later samples (*p* = 0.06). When comparing IL-10 levels in early (*p* = 0.12) and later (*p* = 0.05) samples from fatal and non-fatal cases as opposed to using overall data, differences were not statistically significant. Serum levels of Interleukin-8 (IL-8/CXCL8) and monocyte chemotactic protein-1 (CCL2/MCP-1) were significantly increased in samples from fatal cases and non-fatal cases compared to negative controls, but not between fatal and non-fatal cases when using overall data. Interestingly, three fatal cases (SA979/10, SA1598/10 and SA1448/10) had significantly lower IP-10 levels compared to other fatal cases. Interleukin-6 (IL-6) serum levels were substantially higher (>10 fold) in serum from fatal cases compared to non-fatal cases and negative controls, but the differences were not statistically significant (*p* = 0.13 and 0.1) due to substantial variations in samples from fatal cases. IP10 and IL-6 were also increased in survivors relative to controls, but to lower levels than in fatal cases and not with statistical significance in the case of IL-6. It is noteworthy that chemokine ligand 5 (CCL5/RANTES) was significantly decreased in serum from fatal cases compared to survivors and controls using overall data. Serum samples collected from fatal cases early after onset contained significantly less RANTES than serum from non-fatal cases collected in the same time frame (*p* < 0.0001). The decreased RANTES in later samples from fatal cases compared to non-fatal cases was also statistically significant (*p* = 0.01).

**Table 2:** Chemokine and cytokine concentrations in individual samples studied

Sample ID	Patient gender	Day post onset	Disease outcome	IL-8/ CXCL8 pg/ml	IL-1βpg/ml	IL-6 pg/ml	IL-10 pg/ml	TNF pg/ml	IL-12p70 pg/ml	CCL5/ RANTES pg/ml	CXCL9/ MIG pg/ml	CCL2/ MCP-1 pg/ml	CXCL10/ IP-10 pg/ml	Comments
SPU100/10	M	8	Fatal	735.5	18.3	6526.7	592.4	18.3	13.2	317.9	21788.6	49430.5	25448.3	Hemorrhagic
SPU105/10	M	10	Fatal	1486.7	5.4	379.0	96.3	0.0	0.0	665.1	31437.1	33042.3	23176.3	Hemorrhagic
SPU59/10	M	0	Fatal	10046.3	22.0	8956.6	161.0	7.5	0.0	368.6	19185.9	13389.9	27393.0	Hemorrhagic
SPU97/10	M	7	Fatal	15565.7	12.2	54005.5	915.6	2.3	0.0	486.9	1027.3	11008.3	18799.8	Hemorrhagic
SPU86/10	M	5	Fatal	321.8	2.8	321.2	380.8	6.1	0.0	6249.8	7535.9	6135.8	24694.0	No hemorrhagic syndrome
SPU126/10	F	3	Fatal	1312.9	10.1	1371.7	67.9	9.7	0.0	610.4	2612.9	1176.5	28142.4	Sindbis IgM positive
SPU162/10	F	2	Fatal	585.2	7.0	949.5	83.4	0.0	0.0	285.0	4225.8	758.7	28142.4	Hemorrhagic
SPU115/10	M	0	Fatal	658.7	5.9	476.7	1180.0	0.0	0.0	1325.0	8430.9	1311.4	28335.1	
SPU68/10	M	5	Fatal	193.3	5.6	44.0	16.2	34.8	0.0	114.1	1696.1	529.3	25276.2	Hemorrhagic
SA1372/10	M	3	Fatal	1298.1	5.2	300.9	138.4	5.2	5.2	324.7	8670.0	700.1	12711.7	Hemorrhagic, Sindbis IgM positive
SA1151/10	M	8	Fatal	3320.7	27.7	4873.1	60.6	2.6	5.7	506.2	34137.3	15978.6	21704.5	Hemorrhagic
SA1457/10	M	6	Fatal	385.3	0.0	454.6	110.8	0.0	6.2	2320.6	2790.1	561.5	3829.0	Hemorrhagic; Encephalitis; HIV status unknown
SA979/10	F	7	Fatal	1524.6	7.3	783.2	5.4	0.0	2.2	457.5	2910.1	747.6	766.1	Normal liver enzymes; Hemorrhagic; Sindbis IgM positive
SA1598/10	F	8	Fatal	782.4	4.6	152.7	6.5	0.0	6.2	6043.0	916.4	172.1	323.9	HIV+; encephalitis only; Normal liver enzymes; No hemorrhagic syndrome
SA744/10	M	2	Fatal	433.0	0.0	160.3	28.7	2.2	5.0	367.9	4522.6	1430.6	20833.0	Hemorrhagic; Sindbis IgM positive
SA868/10	M	1	Fatal	543.0	0.0	29.0	133.7	0.0	6.4	7131.7	1429.9	511.8	21451.2	
SA486/10	M	3	Fatal	13809.7	21.1	14055.9	126.0	2.4	7.4	1013.1	56734.4	22156.8	19544.4	
SA1325/10	M	8	Fatal	6503.5	6.8	940.1	173.2	2.7	6.0	4679.1	28687.8	27127.3	20772.2	
SA1448/10	F	5	Fatal	261.8	0.0	39.7	12.0	0.0	5.1	15101.6	392.6	93.6	132.2	Sindbis IgM positive
MEDIAN RESULTS IN FATAL CASES				782.4*	5.9	476.7	110.8*,**	2.3	5.0	610.4***,****	4522.6*,**	1311.4*	21451.2*,**	
SA342/11	M	0	Survivor	303.7	2.6	18.4	0.0	2.3	0.0	24828.0	105.6	1564.9	2754.1	Hospitalized
SA257/10	M	2	Survivor	73.6	0.0	11.9	5.8	0.0	0.0	16970.3	256.3	788.9	5744.4	Hospitalized; Sindbis IgM positive
SA591/10	M	0	Survivor	69.3	2.7	25.8	0.0	0.0	0.0	4397.4	110.7	4934.3	2100.1	Hospitalized
SA921/10	M	1	Survivor	34.0	8.1	27.6	0.0	0.0	0.0	13747.7	236.0	3330.9	17652.1	Hospitalized
SA1601/10	M	7	Survivor	184.0	9.8	967.3	15.1	0.0	0.0	4258.0	84.2	1136.9	668.8	Hospitalized
SA1329/10	M	0	Survivor	150.2	2.8	47.8	0.0	0.0	0.0	3686.7	300.1	13904.5	10918.3	Hospitalized
SPU079/10	M	0	Survivor	295.7	0.0	32.2	0.0	3.2	3.2	6099.1	196.8	353.9	729.3	Hospitalized; WNV and Sindbis IgM positive
SA287/10	M	1	Survivor	3764.8	102.7	3403.8	10.8	171.8	2.4	9180.2	486.3	11250.4	15232.2	Hospitalized
SA226/10	M	2	Survivor	1844.0	19.4	864.8	0.0	58.4	2.7	6815.2	330.6	856.5	3805.3	Hospitalized
SA276/10	M	1	Survivor	1341.5	8.1	370.1	6.7	5.0	0.0	4776.0	329.6	8724.8	7047.2	Hospitalized
SA918/10	M	2	Survivor	4657.3	36.9	8.0	0.0	0.0	0.0	12142.9	203.1	1695.4	614.6	Hospitalized
SA399/10	M	0	Survivor	265.7	0.0	51.4	32.5	0.0	2.8	7936.6	307.3	5832.6	17407.5	Hospitalized
SPU062/10	M	3	Survivor	132.7	3.0	127.8	6.7	0.0	0.0	17843.0	247.2	3465.7	10816.4	
SA165/10	F	3	Survivor	115.9	0.0	10.5	0.0	0.0	0.0	6680.6	104.0	325.9	2923.9	Sindbis IgM positive
SA197/10	M	13	Survivor	296.0	7.6	309.8	70.2	0.0	0.0	4570.4	7520.5	9217.3	17978.2	
SA472/10	M	7	Survivor	499.6	9.4	314.3	0.0	0.0	0.0	8316.3	224.0	3069.1	5206.3	SIndbis IgM positive
SA538/10	M	5	Survivor	54.2	4.9	79.3	0.0	0.0	0.0	10259.9	119.9	2392.9	3561.4	
SA746/10	M	4	Survivor	10789.3	202.8	3228.9	0.0	0.0	0.0	16001.6	138.9	4062.4	1010.0	
SA982/10	F	0	Survivor	469.1	14.9	179.8	13.6	0.0	0.0	16421.8	368.5	3479.0	8817.0	
SA1098/10	M	1	Survivor	41.6	9.0	9.6	0.0	0.0	0.0	18602.3	158.4	238.0	3599.2	
SA327/11	F	2	Survivor	1893.6	8.8	17.2	0.0	0.0	0.0	12504.9	254.7	729.4	2622.1	
SA250/10	M	1	Survivor	77.4	0.0	25.7	0.0	0.0	0.0	6213.1	79.0	490.3	918.4	
SA317/10	M	2	Survivor	148.8	5.8	48.1	0.0	0.0	0.0	5102.8	92.0	449.0	2365.6	
SA093/10	M	2	Survivor	166.3	2.7	26.6	0.0	2.2	0.0	8480.4	372.3	2214.5	10447.3	
SA128/10	M	1	Survivor	20.3	0.0	40.3	0.0	0.0	0.0	19184.5	226.6	2179.7	8363.5	
SA296/10	M	0	Survivor	121.2	5.1	94.2	0.0	0.0	5.5	5810.2	216.9	1849.5	1286.1	
SA325/10	M	2	Survivor	256.8	0.0	9.1	0.0	0.0	4.9	7534.2	135.3	772.8	2357.4	
SA1402/10	M	0	Survivor	136.1	0.0	53.4	0.0	0.0	3.2	5295.1	289.9	4027.0	8440.0	
SPU085/10	M	0	Survivor	175.5	0.0	26.8	0.0	0.0	2.7	8815.6	104.6	1520.0	2786.1	
SPU129/10	M	3	Survivor	171.2	5.5	22.4	0.0	0.0	0.0	19191.9	461.4	626.6	4289.8	
SA139/10	M	2	Survivor	97.9	0.0	17.4	0.0	0.0	5.3	14600.1	217.2	1064.3	4000.9	WN and Sindbis IgM positive
SA494/11	M	3	Survivor	51.5	0.0	7.4	0.0	0.0	2.6	19758.2	85.0	553.5	832.6	
SA523/11	M	5	Survivor	36647.8	921.1	3744.2	0.0	2.7	3.3	19391.7	101.1	539.4	900.0	
MEDIAN RESULTS IN SURVIVORS				171.2*	4.9	40.3	0.0	0.0	0.0	8815.6	217.2	1695.4*	3599.2*	
SPU332/10/28	M	N/A	Control	14.5	6.0	5.7	0.0	0.0	0.0	4606.7	63.2	213.7	9.3	
SPU332/10/9	F	N/A	Control	37.7	0.0	6.9	0.0	0.0	0.0	15593.2	638.6	219.3	135.2	
SPU332/10/27	M	N/A	Control	46.8	6.2	13.1	0.0	0.0	0.0	17658.3	2179.3	501.1	368.2	
SPU332/10/11	F	N/A	Control	218.1	8.9	5.8	0.0	0.0	0.0	15274.5	31.2	37.4	0.0	
SPU332/10/2	M	N/A	Control	202.8	0.0	21.2	0.0	0.0	2.2	14419.2	843.8	601.2	196.2	
SPU332/10/6	M	N/A	Control	28.5	0.0	0.0	0.0	0.0	2.2	24540.3	132.2	45.9	24.0	
SPU332/10/8	F	N/A	Control	37.2	5.0	7.5	0.0	0.0	4.8	17996.2	227.5	20.5	19.3	
SPU332/10/25	M	N/A	Control	9.5	0.0	0.0	0.0	0.0	2.4	54.8	0.0	0.0	0.0	
MEDIAN RESULTS IN CONTROLS				37.5	2.5	6.4	0.0	0.0	1.1	15433.8	179.8	129.8	21.6	

* denotes statistical significant increase relative to results in controls (*p* < 0.01)

** denotes statistical significant increase relative to results in survivors (*p* < 0.01)

*** denotes statistical significant decrease relative to results in controls (*p* < 0.01)

**** denotes statistical significant decrease relative to results in survivors (*p* < 0.01)

Note: because of the 1:4 dilution of serum for the cytokine analysis, a “0 pg/ml” value represents a limit of detection rather than absolute absence of the detected protein

**Fig. 3:**
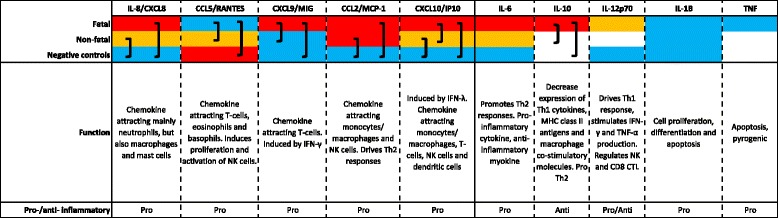
Heatmap showing relative chemokine and cytokine levels between fatal cases, non-fatal cases and negative controls. Relative levels are indicated by color. *Red* indicates a high level, *orange* indicates an intermediate level, *blue* indicates a low level and *absence of color* indicates that no protein was detected. *Brackets* indicate differences which are statistically significant (*p* < 0.01)

**Fig. 4:**
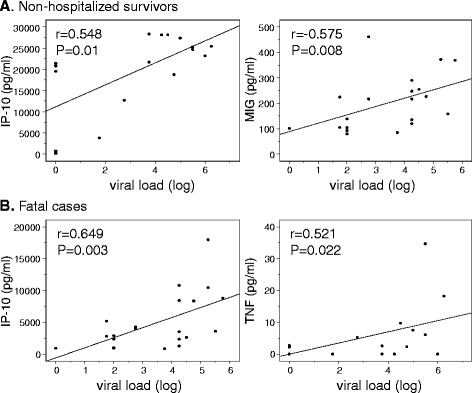
Correlations between viral load and chemokine/cytokine levels. The relationship between serum levels of chemokines/cytokines and viral load (log) was evaluated using the Spearman correlation coefficient. Obvious outliers were excluded from analyses. Only significant correlations (P <0.05) are shown and were observed in non-hospitalized non-fatal cases (**a**) and in fatal cases (**b**). The P values are indicated

Three of the fatal patients had strikingly low concentrations of IP-10 compared to the median of 21451.19 pg/ml in fatal cases. Clinical data were unavailable for one of these cases but interestingly the remaining two did not present with any liver involvement (normal liver enzyme counts). A heat-map diagram showing relative levels of each chemokine or cytokine is shown in Fig. 3, with main functions in the host response to infection. Positive correlations were observed when comparing viral load in fatal cases to IP-10 (*r* = 0.649; *p* = 0.003) and TNF (*r* = 0.521; *p* = 0.022) respectively (Fig. 4). Positive correlations were observed when comparing viral load to IP-10 (*r* = 0.548; *p* = 0.01) and MIG (*r* = 0.575; *p* = 0.008) in a subset of the non-fatal cases (non-hospitalized patients; *n* = 21). No other statistically significant correlations were observed.

## Discussion

The clinical manifestations of severe RVF during the 2010/11 RVF outbreak in South Africa included liver failure, renal failure, thrombocytopenia, encephalitis and hemorrhaging; they are similar to those reported previously [5]. A small number of both fatal and surviving patients had IgM antibodies against other common arboviral diseases in South Africa, including West Nile virus and Sindbis virus. The high percentage of positive HIV status amongst RVF cases associated with encephalitis noted in our study corresponds to earlier observations. A high percentage (89 %) of human cases during the 2007 Tanzania outbreak presented with encephalopathy and all patients with known HIV status, of whom 75 % died, were among this group [6].

It was previously shown that RVFV replicates to significantly higher levels in patients with fatal outcome compared to survivors [32]. Our results point to the same conclusion, but we only found a 1-log_10_ difference in average virus titer (TCID_50_/ml) in serum between fatal and non-fatal cases, compared to a 2.3 log_10_ difference shown in the previous study. This difference is likely due to differences in time of sample collection post disease onset. Samples were collected on average 3 days post onset from both fatal and non-fatal cases in the previous study (ranges 1–7 days from fatal, and 0 to 10 days from non-fatal), compared to 5 days from fatal (range 0–10) and 1 day post onset (range 0–7) from non-fatal cases in this study. The immune response of the host plays a role in the pathogenesis and thus the severity of disease in an infected individual. Dates of disease onset were recorded by healthcare professionals at the hospitals, clinics or general practitioners consulted by patients. Day post onset of blood collection from a number of cases was established as zero (0) due to onset date being recorded as the same date as blood collection. This could likely be attributed to increased awareness of RVF during the outbreak resulting in sick people seeking medical attention immediately upon feeling unwell. One can, however, not rule out incorrect information recorded by professionals or incorrect/inaccurate information supplied by patients, which is a limitation of this study. There was no significant sequence difference between isolates recovered from fatal and non-fatal cases.

The role of the host inflammatory response in RVF disease development has not been extensively studied and most studies have been conducted in laboratory animal models which do not necessarily accurately reflect the response in a host species. Studies in the mouse model revealed a strong involvement of inflammatory and anti-apoptotic responses in lethal outcome [29, 30]. The only previous study in humans concluded that a pro-inflammatory response was required for patient survival after comparing concentrations of soluble factors in serum from six fatal (serum collected average 4.8 days post onset) and 20 non-fatal cases (serum collected average 3.75 days post onset) sampled during the Saudi Arabia outbreak of 2000/01 [31]. Based on results from these previous studies, all pointing towards involvement of the inflammatory response in RVF disease outcome, we investigated the concentration of cytokines involved in inflammation in a larger panel of human serum samples collected from a more recent outbreak to gain more knowledge of this aspect of RVF pathogenesis.

Although, on average, samples were collected later after onset of illness from fatal cases than survivors, the inclusion of data from healthy control individuals serves to normalize the data within the period of acute RVFV infection. To further account for the possible effect of sample time collection, data were compared between fatal and non-fatal cases using the overall dataset, and further grouped according to early and later time points after disease onset. The very low to absent detection of IL-1β and TNF corresponds to the previous study using human samples [31]. Similar to the previous study, we found an increase in the immunosuppressive cytokine IL-10 in fatal cases, while the previous study also found an increase in pro-inflammatory cytokines CXCL1/GRO, which binds to one of the IL-8 receptors CXCR2, and sCD40L in survivors leading the authors to conclude that a pro-inflammatory response is indicative of survival from RVFV infection [31]. In contrast, we found that upregulation of certain proinflammatory cytokines significantly correlates with a fatal outcome, which corresponds to the two previous studies in mice and is likely influenced by the specific cytokines analyzed in the different studies [29, 30].

In this study IL-6 and IL-8, were detected at similar levels in fatal cases and survivors, and were increased when grouped together as RVFV infected individuals (fatal cases and survivors) compared to uninfected individuals (negative controls). Both cytokines can play a role in inflammation, which confirms that, regardless of disease outcome, inflammation is triggered during RVFV infection in humans, as has been reported in other viral hemorrhagic fevers [33–37]. Interleukin-8 (IL-8) is a pro-inflammatory mediating chemokine produced by macrophages in response to infection, while IL-6 is an inflammatory cytokine that also mediates fever. It’s important to note that fatal outcome from Ebola virus disease (EVD) correlates strongly with significantly higher IL-6 and IL-8 compared to non-fatal outcome [38], contrary to our results which show similar levels of both in fatal cases and survivors. However,, EVD has a notably higher fatality rate than RVF and their etiologic agents have different mechanisms by which they counteract host immune responses. A higher level of both cytokines are indicative of liver involvement and damage, as IL-8 has been shown to strongly contribute to liver inflammation [39] while IL-6 is involved in liver regeneration after damage [40].

The following pro-inflammatory chemokines were significantly increased in fatal cases when compared to survivors and negative controls: CXCL9/MIG and CXCL10/IP-10; or in fatal cases and survivors compared to negative controls: CCL2/MCP-1. The chemokines CXCL9 and CXCL10 bind to the same receptor CXCR3. These results correspond to observations on other viral hemorrhagic fevers (VHFs) such as hemorrhagic fever with renal syndrome (HFRS), Severe Fever with Thrombocytopenia (SFTS), Crimean-Congo hemorrhagic fever (CCHF) and Ebola virus disease (EVD) [33–38, 41]. The proinflammatory chemokines are involved in the mobilization of various immune cells to the site of infection that include natural killer cells, dendritic cells, and monocytes/macrophages. It is also interesting that three fatal cases that had very low levels of IP-10 compared to other fatal cases (between 27 and 60 fold lower compared to median for the fatal group), which corresponded to absence of liver failure in two (detailed clinical information not available for the third). The positive correlation between viral load and IP-10 found in fatal cases and non-fatal cases (non-hospitalized) suggests that high viral load possibly contributed to liver damage in the individuals in this study.

It is noteworthy that the immunosuppressive anti-inflammatory IL-10 was also increased in fatal cases versus survivors and negative controls, which suggests an imbalance in inflammatory response, however increased IL-10 is a common occurrence in patients infected with VHF pathogens mentioned above. Interestingly the serum concentration of CCL5/RANTES, a chemokine also involved in chemotaxis of lymphocytes, monocytes, and eosinophils, was significantly decreased in fatal cases compared to survivors and negative controls. RANTES plays a role in the activation and proliferation of T-cells and is thus pro-inflammatory, further pointing towards the dysregulation of the inflammatory response during RVF infection. RANTES levels have been shown to be low in severe and fatal cases of bacterial sepsis, meningococcal disease, Chikungunya fever and cerebral malaria in children [42–45]. Two studies have shown similar levels of RANTES during infection with Ebola and Sudan viruses, when comparing fatal to non-fatal cases [37, 38], while a recent study found increased RANTES levels in surviving pediatric Ebola virus disease patients [46]. RANTES is released by a variety of cell types, including platelets [47–50] which play a major role in blood clotting. From the limited number of fatal cases for which more detailed clinical information was available (*n* = 18), four did not display hemorrhagic manifestations. Two of these were not included in our cytokine analysis due to insufficient sample volume. However, we noted that the remaining two non-hemorrhagic cases, which were included in the cytokine analysis, had strikingly higher levels of RANTES (10 fold at 6249.81 pg/ml and 6042.98 pg/ml) compared to other fatal cases (median 610.4 pg/ml). Although statistically weak in terms of sample numbers, this result is interesting and might point towards an association of low RANTES and hemorrhagic manifestation of RVF. No correlation was observed between platelet counts and RANTES levels (R^2^ = 0.005) within the fatal case group and platelet counts were not available for non-fatal cases.

When the fatal and non-fatal datasets were grouped according to early and later after disease onset, only two cytokine/chemokine concentration differences remained statistically significant; RANTES (early and later after disease onset) and IP-10 (early after onset). The loss of statistical significance of factors previously identified as significant in the overall groups is likely due to the decreased sample numbers once data were stratified according to time. A very strict alpha (α) value of 0.01 was also used as opposed to the usual 0.05 to ensure robustness of statistical conclusions.

A number of the cytokines and chemokines that were raised or decreased in fatal cases versus survivors and negative controls, were in turn also significantly, albeit to a much lesser extent, raised or decreased when comparing RVF survivors to negative controls. This indicates that a certain level of inflammation is normally elicited by RVFV infection, even when the host is able to overcome the infection, but that the inflammatory response in these surviving individuals is much less severe and at a more appropriate level, or that the ability to terminate inflammatory responses were less effective in fatal cases. Inflammatory cytokines and chemokines form an integral part of the innate response to infection, responsible for recruiting and activating various immune cells and acting as co-stimulatory signals to induce adaptive immunity. Therefore a certain level of elevated cytokines is required for a host to mount an efficient immune response to infection. The balance and level of these cytokines seem to be very important in RVF, with dysregulation likely contributing to a fatal outcome. The underlying host factors that might contribute to either a well-controlled or uncontrolled inflammatory response during RVFV infection and other hemorrhagic fever virus infections remains to be investigated. These differential responses and outcomes, aside from the extent of viral exposure, are likely influenced by host genetics, lifestyle, diet, underlying conditions or medication.

## Conclusions

An uncontrolled inflammatory response to RVFV infection correlated with a fatal outcome in certain individuals infected with the virus during the 2010/11 RVF outbreak in South Africa. The cytokines identified in our study could be used as indicators of fatal outcome, but more importantly could help focus efforts on treatment therapies, possibly those aimed at decreasing the effects of an aberrant inflammatory response.

## Methods

### Patient samples

During the 2010/11 RVF outbreak in South Africa, human serum samples from suspected RVF cases were submitted to the Centre for Emerging and Zoonotic Diseases, National Institute for Communicable Diseases (NICD-NHLS), for laboratory confirmation, which included screening for other arboviral infections [51]. Of a total of 278 laboratory confirmed cases, 104 sera were analyzed in the study, of which 19 were from fatal, and 85 from non-fatal cases. Sera were separated from clotted blood and aliquoted for routine diagnostic testing and −70 °C storage for follow-up testing. Ethical clearance for the use of the samples was obtained from the University of the Witwatersrand Human Research Ethics Committee (HREC), clearance number M120239.

### Virus titration

Titration of virus in human serum was performed as described previously [52] by preparing quadruplicate tenfold dilutions in culture medium on microtiter (96-well) tissue culture plates, addition of Vero cells and incubation at 37 °C in 5 % CO_2_ incubator for up to 14 days. Cytopathic effects (CPE) were recorded and virus titers calculated by the Kärber method [53] were expressed as median tissue culture infectious dose (TCID_50_) per ml of serum. Only serum samples of sufficient volume, and from which virus was isolated in suckling mice and/or viral RNA detected by RT-PCR during the diagnostic process, were subjected to TCID_50_ titration (*n* = 98 serum samples).

### Whole genome sequencing of RVFV isolates

A total of 12 viral isolates (first passage on Vero cells) were selected for whole genome sequencing, consisting of six isolates from fatal cases and six isolates from non-fatal cases. Vero cells were infected with human serum and grown until 80–90 % CPE. To avoid accumulation of misleading mutations in the virus genome during in vitro passaging of virus in Vero cells, we sequenced first passage Vero isolates. Supernatants (SNF) were harvested, clarified of cellular debris and RNA extracted from 140 μl SNF using the QIAamp viral RNA kit (Qiagen, Germany). RVF genome specific primers described previously [54] were used to amplify whole S and M segments and two overlapping fragments of the L segment using a Titan One step RT-PCR kit (Roche, Germany). RT-PCR products were cleaned up using a Promega Wizard PCR clean up system (Promega, USA). Sequencing was outsourced to the Stellenbosch University Central Analytical Facilities (SUN-CAF) and performed as follows. Purified amplicons were barcoded and sequenced using 316 Chips with 200 bp chemistry on an Ion PGM™ system (Life Technologies, USA). Analysis of sequence data were performed using the Torrent Suite software. Reads were mapped to Genbank consensus sequence (NCBI BioProject PRJNA14631) using Mira version 3.9.18 (http://mira-assembler.sourceforge.net/) to obtain a FASTA file.

### Serum cytokine quantification by Cytometric Bead Array (CBA)

A total of 60 serum samples were selected for CBA analysis. This total consisted of 19 serum samples from fatal cases, 33 serum samples from non-fatal cases and eight serum samples from uninfected persons (healthy controls) sampled in a post-outbreak survey. The samples from infected patients (fatal and non-fatal) consisted of a selection of sera virologically or serologically laboratory confirmed cases. Available sample volume was a limiting factor with regards to the selection of samples for this particular analysis.

All serum samples were tested in duplicate at 1:4 dilution and CBA kits designed to measure specific sets of cytokines were used. The BD CBA Human Chemokine kit (Becton, Dickinson and Company, USA) was used to quantitatively measure interleukin-8 (IL-8), RANTES (CCL5), monokine induced by interferon-gamma (CXCL9/MIG), monocyte chemoattractant protein-1 (MCP-1) and interferon-gamma-induced protein-10 (IP-10). The BD CBA Human Inflammatory Cytokines kit (Becton, Dickinson and Company, USA) was used to quantitatively measure IL-8, interleukin-1β (IL-1β), interleukin-6 (IL-6), interleukin-10 (IL-10), tumor necrosis factor (TNF) and interleukin-12p70 (IL-12p70). Assays were performed according to the manufacturer’s instructions. Flow cytometry was performed using a dual-laser BD FACSCalibur™ system (BD Biosciences, USA) utilizing BD CellQuest™ software for acquisition. Dilution series of human cytokine standards, included in each kit and prepared according to the manufacturer’s instructions, were included in each assay run to enable quantification. Data were analyzed using FCAP Array software v1.0. Note IL-8 was analyzed with both kits and although concentrations of the cytokine differed slightly due to the difference in preparation steps between the kits, the outcome of fatal versus non-fatal and healthy controls were the same, thus only results from the Chemokine kit is shown.

The relationship between serum levels of cytokines and viral load (log) was evaluated using the Spearman correlation coefficient. Obvious outliers were excluded from analyses.

### Statistics

Equality of variance of cytokines/chemokines between fatal and non-fatal cases was calculated for each cytokine/chemokine analyzed using the F-test in Microsoft Excel Analysis ToolPak. Confidence intervals and *p*-values (two sample *T*-test assuming either equal or unequal variance depending on the outcome of the F-test) were calculated using Microsoft Excel Analysis ToolPak. The alpha (α) value was set to 0.01 to ensure high confidence in statistical significance. Comparison of viral load and cytokine levels was performed using the Spearman correlation coefficient. SPSS software (SPCC Inc., Chicago, Illinois) were used for these statistical analyses.
